# Supplementary material for: Evaluation for the Genetic Association between Store-Operated Calcium Influx Pathway (STIM1 and ORAI1) and Human Hepatocellular Carcinoma in Patients with Chronic Hepatitis B Infection
Source: Biology (Basel). 2020 Nov 9;9(11):388. doi: 10.3390/biology9110388 (PMC7695319; doi:10.3390/biology9110388)
Supplement: Supplementary file 1 [file biology-09-00388-s001.pdf]

Supplementary Materials

Lalu Muhammad Irham [1,2], Wan-Hsuan Chou [1,3], Yu-Shiuan Wang [4,5], Wirawan Adikusuma[1,6], Henry Sung-Ching Wong [1,3], Dyah Aryani Perwitasari [2], Wan-Chen Huang [4], Ben-Kuen Chen [7,8,*], Hwai-I Yang [9,10*], Wei-Chiao Chang [1,3,11,12,*]

1 Department of Clinical Pharmacy, School of Pharmacy, College of Pharmacy, Taipei Medical University, Taipei 11031, Taiwan
2 Faculty of Pharmacy, University of Ahmad Dahlan, Yogyakarta 55164, Indonesia
3 Master Program for Clinical Pharmacogenomics and Pharmacoproteomics, School of Pharmacy, Taipei Medical University, Taipei 11031, Taiwan
4 Institute of Cellular and Organismic Biology, Academia Sinica, Taipei 115, Taiwan
5 Ph.D Program in Clinical Drug Development of Chinese Herbal Medicine, College of Pharmacy, Taipei Medical University, Taipei 11031, Taiwan
6 Departement of Pharmacy, University of Muhammadiyah Mataram, Mataram 83127, Indonesia
7 Ph.D. Program for translational Medicine, College of Medical Science and Technology, Taipei Medical University, Taipei 11031, Taiwan
8 Department of Pharmacology, College of Medicine, National Cheng Kung University, Tainan 701, Taiwan
9 Genomics Research Center, Academia Sinica, Taipei 115, Taiwan
10 Institute of Clinical Medicine, National Yang-Ming University, Taipei 112, Taiwan
11 Integrative Therapy Center for Gastroenterologic Cancers, Wan Fang Hospital, Taipei Medical University, Taipei 116, Taiwan
12 Department of Medical Research, Shuang Ho Hospital, Taipei Medical University, New Taipei City 23561, Taiwan
* Correspondence: bkchen58@mail.ncku.edu.tw (B.-K.C.); hiyang@gate.sinica.edu.tw (H.-I.Y.); wcc@tmu.edu.tw (W.-C.C.)

Contents:

Supporting Information Tables

Table S1. Associations between *STIM1* haplotypes and hepatocellular carcinoma (HCC) in hepatitis B virus (HBV) patients
Table S2. Association between *ORAI1* haplotypes and hepatocellular carcinoma (HCC) in hepatitis B virus (HBV) patients
Table S3. Expression Quantitative trail loci (eQTL) analysis from Genotype-tissue expression (GTEx).

Supporting Information Figures

Figure S1. The linkage disequilibrium plot of the *STIM1* gene.
Figure S2. The linkage disequilibrium plot of the *ORAI1* gene.
Figure S3: Correlation between genotypes and expression level in tissues determined by *Cis*-expression quantitative trait loci (*cis*-eQTLs).

Supplementary Table 1. Association between *STIM1* haplotypes and hepatocellular carcinoma (HCC) in hepatitis B virus (HBV) patients

Haplotype	Frequency		p value	q value
	Case[a]	Control[b]		
**Block 3**: rs11030209/ rs10835270/ rs11030210/ rs7929653/ rs6578418/ rs10835272/ rs10742189				
A/G/G/G/C/A/A	0.231	0.226	0.7364	0.8426
A/G/G/A/C/G/A	0.168	0.158	0.5272	0.8426
C/T/G/A/C/G/C	0.159	0.151	0.5677	0.8426
A/G/G/G/C/G/A	0.126	0.131	0.7349	0.8426
A/G/G/G/C/A/C	0.061	0.072	0.2828	0.8426
C/T/G/G/C/G/C	0.057	0.042	0.0836	0.8426
A/G/T/A/G/A/C	0.032	0.027	0.4395	0.8426
A/G/G/A/C/A/A	0.021	0.028	0.3342	0.8426
A/G/G/G/C/G/C	0.021	0.026	0.4307	0.8426
C/T/G/A/C/A/C	0.013	0.019	0.3192	0.8426
A/G/G/A/C/G/C	0.016	0.014	0.7236	0.8426
A/G/G/A/C/A/C	0.006	0.013	Reference	
**Block 6**: rs7924984/ rs2412338/ rs10835402/rs11030472/ rs11030478/ rs11030486/ rs727152/rs2959081				
G/G/T/A/G/C/A/T	0.039	0.024	**0.0163**	0.3097
A/T/C/G/G/C/A/T	0.224	0.196	0.0711	0.8426
A/T/T/A/A/T/A/T	0.195	0.192	0.8426	0.8426
G/G/T/A/G/C/G/T	0.095	0.103	0.5102	0.8426
A/T/C/A/G/C/A/T	0.077	0.084	0.565	0.8426
G/G/T/A/G/C/A/C	0.364	0.388	Reference	
**Block 7**: rs11030639/ rs7116520/ rs1442725/ rs11030841/rs4910882				
A/G/C/G/G	0.366	0.413	**0.015**	0.3000
G/A/C/G/G	0.330	0.305	0.1734	0.8426
A/A/C/A/A	0.223	0.208	0.3784	0.8426
A/A/T/A/A	0.059	0.051	0.3147	0.8426
A/A/C/G/G	0.010	0.013	Reference	

[a] HBV with HCC. [b] HBV without HCC. Significant (p<0.05) values are in **bold.** The q value is the false discovery rate (FDR) estimation for multi-testing.

Supplementary Table 2. Association between *ORAI1* haplotypes and hepatocellular carcinoma (HCC) in hepatitis B virus (HBV) patients

Haplotype	Frequency		*p* value	q value
	Case[a]	Control[b]		
**Block 1**: rs6486795/ rs74936888/ rs3741595				
T/T/C	0.670	0.621	**0.013**	0.2990
C/T/T	0.238	0.269	0.073	0.8426
C/C/C	0.058	0.079	0.047	0.8426
C/T/C	0.035	0.030	Reference	

[a] HBV with HCC. [b] HBV without HCC. Significant (p<0.05) values are in **bold.** The q value is the false discovery rate (FDR) estimation for multi-testing.

Supplementary Table S3. Expression Quantitative trail loci (eQTL) results of The SNP from Genotype-tissue expression (GTEx).

SNP ID	Gencode ID (ENSG00000-)	Gene symbol	p value	Effect size	Tissue	Actions
rs7116520	167323.11	*STIM1*	1.1e-9	-0.18	Nerve - Tibial	GG>AG>AA
	167323.11	*STIM1*	2.0e-8	-0.22	Heart-Left Ventricle	GG>AG>AA
rs6578418	NA	NA	NA	NA	NA	NA
rs11030472	NA	NA	NA	NA	NA	NA
rs6486795	276045.2	*ORAI1*	1.30E-17	0.22	Whole Blood	CC>CT>TT
	276045.2	*ORAI1*	9.90E-16	0.29	Esophagus - Mucosa	CC>CT>TT
	276045.2	*ORAI1*	3.30E-13	0.23	Thyroid	CC>CT>TT
	276045.2	*ORAI1*	7.60E-08	0.24	Stomach	CC>CT>TT
	276045.2	*ORAI1*	4.50E-07	0.25	Pancreas	CC>CT>TT
	276045.2	*ORAI1*	4.80E-07	0.11	Muscle - Skeletal	CC>CT>TT
	276045.2	*ORAI1*	1.5E-06	0.21	Colon - Transverse	CC>CT>TT
	276045.2	*ORAI1*	0.000029	0.19	Esophagus - Gastroesophageal Junction	CC>CT>TT
	276045.2	*ORAI1*	0.000058	0.17	Heart - Atrial Appendage	CC>CT>TT
	276045.2	*ORAI1*	0.00016	0.13	Esophagus - Muscularis	CC>CT>TT

Source: Expression Quantitative trail loci (eQTL) obtained from https://gtexportal.org/home, NA: Not Available

Supplementary Figure

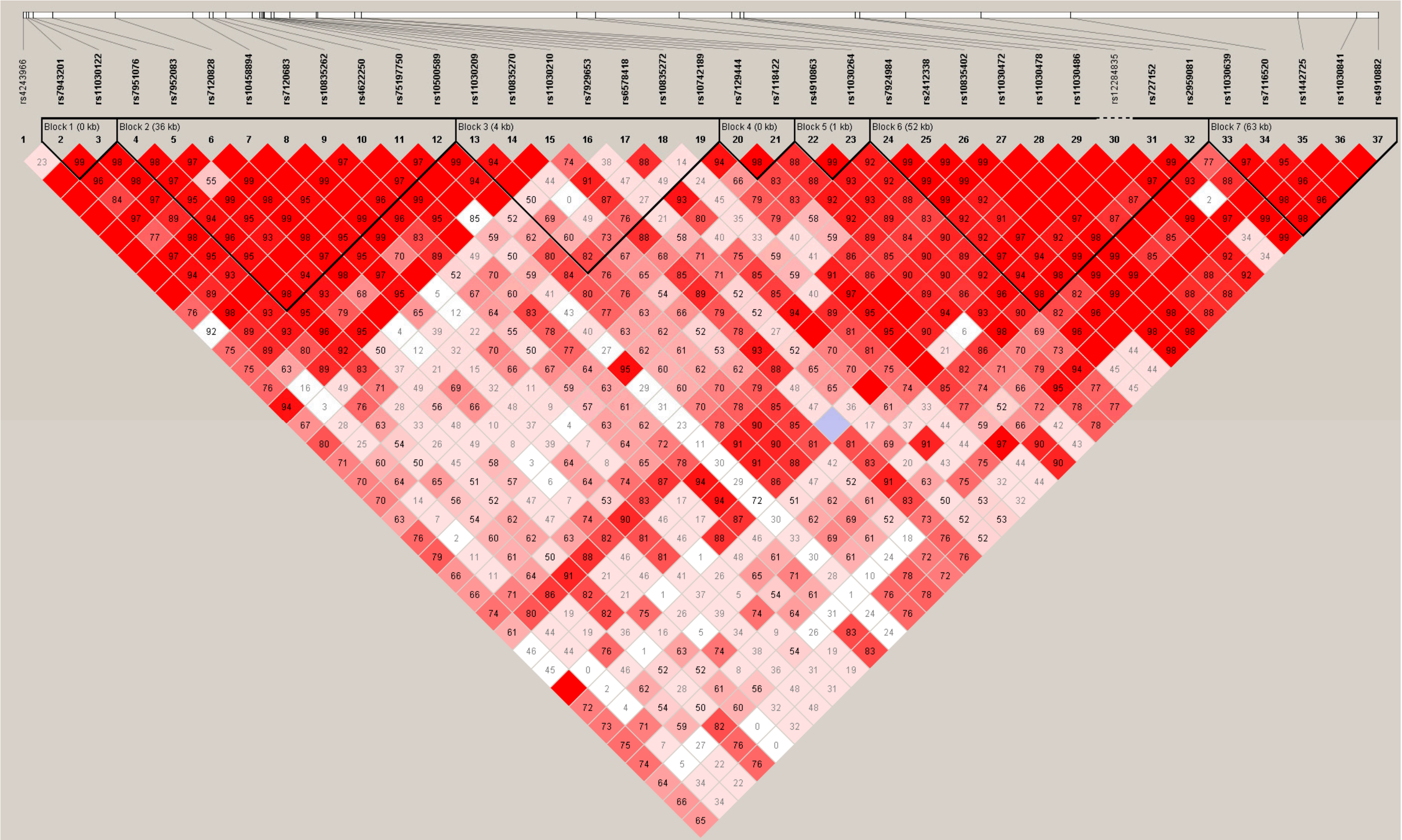

Based on D-prime, D'

Supplementary Figure 1. Linkage disequilibrium plot of the *STIM1* gene.

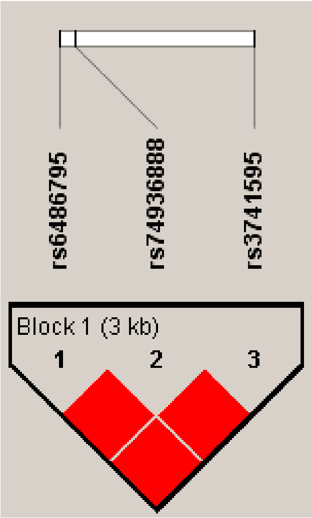

Based on D-prime, D'

Supplementary Figure 2. Linkage disequilibrium plot of the *ORAI1* gene.

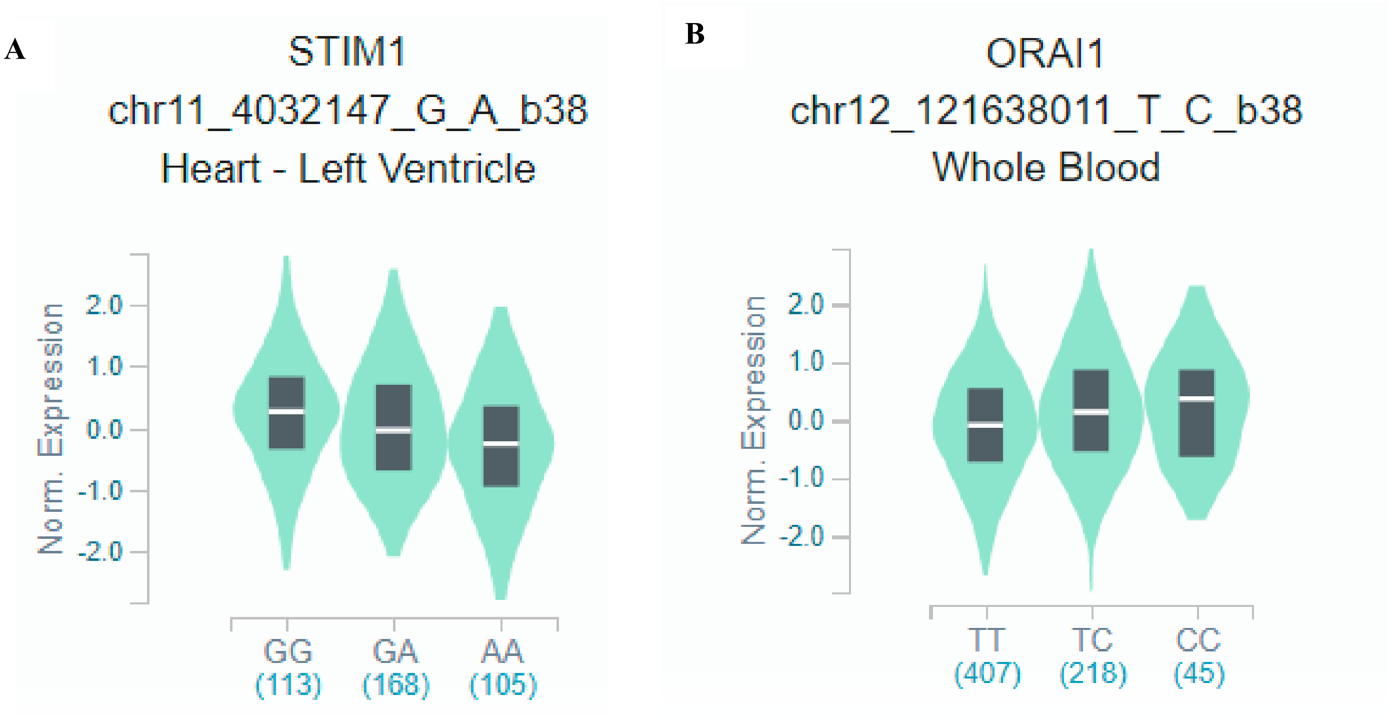

Figure S3. Correlation between genotype and expression in tissue determined by *Cis*-expression quantitative trait loci (*cis*-eQTLs). Figure **A** the expression of *STIM1* between rs7116520 Homozygous (GG), Heterozygous (AG), and Homozygous (AA) in heart-left ventricle tissue. Figure **B** the expression of *ORAI1* between rs6486795 Homozygous(CC), Heterozygous (TC) and Homozygous (TT) in whole blood.

.
